# Supplementary material for: Pregnancy outcomes of women whom spouse fathered children after tyrosine kinase inhibitor therapy for chronic myeloid leukemia: A systematic review
Source: PLoS One. 2020 Dec 3;15(12):e0243045. doi: 10.1371/journal.pone.0243045 (PMC7714354; doi:10.1371/journal.pone.0243045)
Supplement: Attachment — Submitted filename: Rebuttal letter.docx [file pone.0243045.s001.docx]

**Rebuttal letter**

**Reviewer #1:** In this manuscript Szakacs and colleagues have conducted a systematic review of literature assessing the association between TKI therapy and pregnancy outcomes in CML male patients.

The authors performed diligent systematic research using 7 electronic databases and found 35 potentially eligible manuscripts. Final 25 manuscripts were included in the review (after removal of over lapping study populations.

Of the 362 pregnancies studies, 320 pregnancies were fathered w/o TKI discontinuation while 345 ended in live births. 10 pregnancies (2.9%) ended up in fetal malformations. This is comparable with the rate of malformations in the general population. Moreover the rate of malformations noted were highest with nilotinib. I agree with the authors that this info should be used with caution since only 20 live births were on patients taking nilotinib. The authors also found no impact of continuing TKI therapy at the time of conception and thereafter on the rate of malformation.

This is an overall well written manuscript. I recommend it to be accepted.

**[Authors’ comment]:** We appreciate the kind words. Thank you for the time spent reviewing our paper.

**Reviewer #2:** Overall, this is a well-conducted systematic review on an important topic that often seems overlooked. A few comments that I think may strengthen the manuscript:

**[Authors’ comment]:** We greatly appreciated the comments, which, we believe, have significantly improved the manuscript.

1) I think the title can be revised - “Pregnancy outcomes of males...” sounds as though the man is carrying the child. Perhaps something along the lines of “Males who father children on TKIs” or “offspring of men treated with TKIs”

**[Authors’ reply]:** Indeed, our title may be misunderstood. We rephrased it to ’Pregnancy outcomes of women whom spouse fathered children after tyrosine-kinase inhibitor therapy for chronic myeloid leukemia: A systematic review’.

2) Similarly, I think there are several areas in the manuscript where the English is imprecise, or sentences are incomplete (fragments) - for example, page 21, line 300 “Nor there was the analysis of dose-response” is a fragment, as is page 22 line 319: “Not to mention the lack of detailed, long-term follow-up data on the offspring...”

**[Authors’ reply]:** We revised the manuscript and attempted to improve the English language.

3) Table 2: In the table it seems that the numbers skip from 1 (Abruzzese, under “infant complications”) to 4 (Carlier, under “No of pregnancies”).

**[Authors’ reply]:** We apologize for the oversight. Now in Table 2, superscript numbers read in ascending order from left to right and top to bottom. Besides, footnotes and abbreviations were simplified to ease understanding.

4) If available, on page 12 under “Planned treatment discontinuation”, some information on low long TKI was discontinued would be helpful to have. Did these patients stop TKI 1 week before trying to conceive? 6 months?

**[Authors’ reply]:** Thank you for the idea. We added a column to Table 1, where we report these data. The time intervals ranged between 15 days to 5 months.

5) For figure 2, the various shades of blue are VERY hard to distinguish. In addition, I think fig 2B and 2D should be omitted - the information is available in the text and is not difficult to comprehend, so I do not think these two figures are much of a visual aid.

**[Authors’ reply]:** We removed Figs 2B and 2D, which resolved the issue with the hard recognition of the various shades of blue.

6) Reasons for elective termination of pregnancies should be added, if available. Although numbers are obviously limited, this information may shed some light on other consequences of TKI treatment and fathering children.

**[Authors’ reply]:** We revised the papers for the information. Unfortunately, only Mukhopadhyay et al. 2015) reported the cause of elective termination in the case of an unplanned pregnancy.

7) Regarding the discussion, page 20, 2nd full paragraph (lines 272-286) is somewhat repetitive as this information is mostly covered in the introduction. If the authors elect to keep this paragraph, it seems out of place - it would probably fit better earlier in the discussion, maybe as the first paragraph.

**[Authors’ reply]:** We agree on the point so that almost the entire content of the paragraph mentioned has been removed.

8) The first several sentences of the 3rd full paragraph on page 20, starting with “If a couple desires fertility...” is not based on the data that the authors present in this manuscript, nor on data referenced in the literature. If recommendations are to be made based on the authors’ opinions, this should be in the “implications for clinical practice” paragraph, and should clearly state that these are based on the authors’ experience/opinion.

**[Authors’ reply]:** Fair point. The paragraph mentioned has been removed. The concept of semen cryopreservation before TKI treatment, as an alternative of planned treatment discontinuation, is described in the “implications for clinical practice” paragraph.

9) Lastly, the section on “limitations of the evidence” in the discussion is much too long and the authors could afford to be more concise here.

**[Authors’ reply]:** The section mentioned was shortened from 393 to 208 words.
